# Into the fourth dimension

**DOI:** 10.7554/eLife.31328

**Published:** 2017-10-04

**Authors:** David L Des Marais

**Affiliations:** Department of Civil and Environmental EngineeringMassachusetts Institute of TechnologyCambridgeUnited States

**Keywords:** *Brassica rapa*, photosynthesis, transcriptomic network analysis, drought, abiotic stress, daily rhythms, Other

## Abstract

The influence of time on the drought response of *Brassica rapa*, an agriculturally important species of plant, has been clarified.

**Related research article** Greenham K, Guadagno CR, Gehan MA, Mockler TC, Weinig C, Ewers BE, McClung CR. 2017. Temporal network analysis identifies early physiological and transcriptomic indicators of mild drought in *Brassica rapa*. *eLife* **6**:e29655. doi: 10.7554/eLife.29655

Shortages of water and variations in temperature are probably the biggest constraints on the yields of crops in agriculture worldwide (Boyer, 1982). Drought can affect nearly every process in a plant, from energy production to growth, and plants respond to drought stress through a variety of complex mechanisms (Levitt, 1980). These responses often begin at a molecular level, but despite decades of research, it remains surprisingly challenging to link specific molecular processes to more visible signs of stress in a plant. This means that we are unable to answer some basic questions. Why do stressed plants die (McDowell et al., 2008)? And how does drought stress affect photosynthesis and the ‘carbon budget’ of plants (Pinheiro and Chaves, 2011)?

In recent decades, there has been major progress in the use of genetic engineering to make crops resistant to pests, but efforts to develop drought-tolerant crops have been less successful (Passioura, 2010). This could have several reasons, the complex responses of plants to drought stress being one. Conceptual and technical challenges, including the disagreement about how to experimentally ‘stress’ a plant in the first place, contribute to our lack of understanding (Blum, 2016). Now, in eLife, C. Robertson McClung and co-workers – including Kathleen Greenham and Carmela Rosaria Guadagno as joint first authors – report results that ease my own concerns about how to best address these challenges (Greenham et al., 2017).

Greenham et al. exposed *Brassica rapa*, which is both an important crop species and a widely-studied model system, to a gradually increasing level of mild drought over four days, and measured how the plants responded during the final 48 hours of the treatment. This gradual exposure to stress is very similar to what plants experience in the field (for example, in the days following a rain fall, or when a center-pivot irrigator slowly revolves through a field). Moreover, the researchers – who are based at Dartmouth College, the Donald Danforth Plant Science Center and the University of Wyoming – measured traits that matter, rather than traits that are easy to measure (Passioura, 2010). Their key contribution, however, is in exploring the fourth dimension of the stress response – time.

Time plays two important roles in the study. First, the plants respond more strongly as their soil progressively dries out: Greenham et al. see a clear indication of this in their physiological and molecular data. Second, the sampling protocol includes a 24-hour, or diel, design in which traits are measured around the clock for two days. This is necessary because the rate of water use by a plant varies over the course of a 24-hour period: plants use water in photosynthesis during the day, and also lose more water due to evaporation because day-time temperatures are higher than night-time temperatures. These daily fluctuations also interact with the circadian clock of the plant (Harmer, 2009). The approach used by Greenham et al. gives them the statistical power they need to identify correlations between molecular and whole-plant processes (Figure 1).

**Figure 1.**
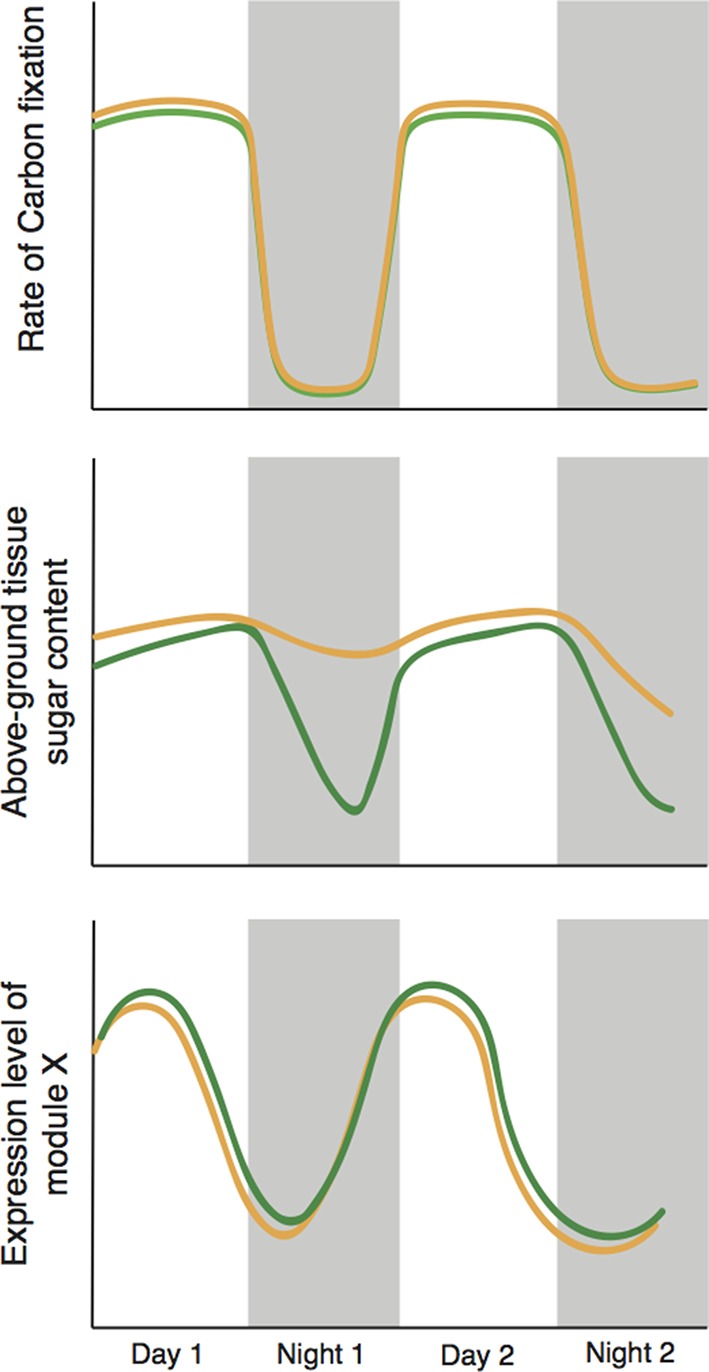
Cyclic behavior in the plant *Brassica rapa* (schematic). Greenham et al. exposed the plants to mild drought over four days, with the level of drought slowly increasing over time, and measured a number of traits over the last 48 hours; results for well-watered plants are shown in green, drought-stressed plants are shown in brown. Mild drought had a negligible effect on the rate of photosynthetic carbon reduction (top). However, mild drought led to increased leaf sugar content overnight (middle); it is possible that this helps to maintain favourable 'water relations' between cells and the external environment. The presence of two types of sample variation in the experiment – the increase in the level of drought with time, and the natural circadian cycle – allowed Greenham et al. to identify the genes that respond to drought (bottom). They did this by identifying genetic modules with levels of expression that correlate with variations in the rate of carbon fixation over time, and differ slightly in well-watered plants (green) and plants exposed to mild drought (brown).

What have we learned? The first important finding is practical. The activity of genes varies more with time of day (an effect due to the circadian clock) than the gradual increase in drought stress applied by the researchers. Given the enormous effects that circadian processes have on cell biology, this is perhaps unsurprising. But the strong influence of time of day on gene expression needs to be taken into account in experiments of this type.

The second important finding relates to plant carbon budgets. It is commonly assumed that drought-stressed plants close their pores – or stomata – to preserve water, thereby reducing their photosynthetic capacity and also reducing growth (Shinozaki and Yamaguchi-Shinozaki, 2007). However, Greenham et al. clearly demonstrate that the photosynthetic ability of stressed *B. rapa* plants remains high – in fact, sugar levels in the leaves are higher than in control plants. Moreover, by cleverly integrating molecular and whole-plant data using tools from network theory and patterns of changes over time, they were able to identify groups of genes that may drive these processes and effects.

Where does the field go from here? To my mind, the next step should be to use a similar experimental approach to analyze other varieties of *B. rapa*. To breed useful response traits requires genetic diversity in response (Des Marais et al., 2013). Once potential differences in response have been identified, it may be possible to manipulate and modify the relevant genes. These will be challenging experiments, but Greenham et al. have given us a blueprint for moving the field forward.
